# Distribution pattern of medial group retropharyngeal lymph nodes and its implication in optimizing clinical target volume in nasopharyngeal carcinoma

**DOI:** 10.3389/fonc.2023.1228994

**Published:** 2023-09-05

**Authors:** Dan Zong, Ning Jiang, Cheng Kong, Jing Wen, Li-jun Wang, Ye-song Guo, Lan-fang Zhang, Xia He, Zhen-zhang Chen, Sheng-fu Huang

**Affiliations:** ^1^ Department of Radiation Oncology, Jiangsu Cancer Hospital, Jiangsu Institute of Cancer Research, Nanjing Medical University Affiliated Cancer Hospital, Nanjing, Jiangsu, China; ^2^ Department of Medical Imaging, Jiangsu Cancer Hospital, Jiangsu Institute of Cancer Research, Nanjing Medical University Affiliated Cancer Hospital, Nanjing, Jiangsu, China

**Keywords:** nasopharyngeal carcinoma, the medial group of retropharyngeal lymph nodes, diffusion-weighed imaging, Lasso regression analysis, swallowing structures

## Abstract

**Purpose:**

This study aimed to determine the diagnostic value of diffusion-weighted imaging (DWI) and to elucidate the clinical characteristics of medial group retropharyngeal lymph nodes (RLNs) based on multi-modal imaging. Also, we intended to explore the feasibility of optimizing the CTV60 boundary based on the characteristics of medial group RLNs.

**Methods:**

A total of 549 patients with nasopharyngeal carcinoma received magnetic resonance imaging (MRI), DWI, and contrast-enhanced computed tomography (CT) to detect and evaluate clinical characteristics of medial group RLNs. ^[18F]^Fluorodeoxyglucose positron emission tomography/computed tomography was utilized to identify fluorodeoxyglucose uptaking and contrast-enhanced CT to ensure the reliability of CTV optimization during radiotherapy. The DESdC (Drinking, Eating, Swallowing Difficulties, and Coughing while Eating or Drinking) score was utilized to evaluate swallowing disability.

**Results:**

Fourteen of 549 patients had medial group RLNs with a transverse diameter of 2.0–19.0 mm, which distributed between the upper margin of 1st cervical vertebra (C1) and the upper one-third of C3. Lasso regression and Pearson chi-square test suggested that its occurrence was associated with stage N, bilateral cervical lymph node metastases, especially when the transverse diameter of cervical lymph nodes was > 3 cm. The sensitivity of DWI, T2 STIR, and contrast-enhanced CT was 100%, 57.1%, and 21.4%, respectively. We optimized CTV60 of medial group RLNs from the base of skull to the upper edge of C2 excluding specific cases. For patients with CTV60 optimization, radiation dose and volume of swallowing structures decreased obviously. Based on our radiotherapy strategy on CTV60, acute toxicities of enrolled patients were well tolerated. Ninety-six of 549 patients had scores with DESdC score. Eighty-three patients scored 1, seven patients scored 2, one patient scored 3, and three patients scored 4. The median interval from the onset of symptoms was 72 (4–114) months. The 5-year overall survival, progression-free survival, local recurrence-free survival, and distant metastasis-free survival were 87%, 80%, 93%, and 85%, respectively. None of the patients with regional recurrence happened in the optimized region.

**Conclusion:**

DWI possesses superiorities in displaying lymph nodes. Based on the low incidence of the medial RLNs, CTV60 of medial group RLNs from the base of skull to the upper edge of C2 is feasible and has dosimetric advantages for protecting swallowing structures.

## Introduction

The prevalence of nasopharyngeal carcinoma (NPC) varies significantly by area and is particularly high in southern China (1). Intensity-modulated radiotherapy (IMRT) is widely used as the mainstay therapeutic modality due to its biological behavior and radiosensitivity (2). IMRT offered better target conformity and lower doses to surrounding critical organs compared with two- or three-dimensional radiotherapy. With applications of improved radiotherapy techniques and administration of chemotherapy, treatment outcomes and quality of life have been greatly improved in NPC patients (3, 4). However, the high radiotherapy dose and extensive volume coverage determined by particular anatomical position and locoregionally advanced disease result in high incidences of acute mucosal reaction and long-term dysphagia, xerostomia, and cervical fibrosis (5).

As patients survive longer, long-term adverse effects caused by radiotherapy become apparent and a significant factor affecting patients’ quality of life. We have initiated a series of studies to explore injuries of essential organs such as the brainstem and temporal lobe after standard treatments according to the protocol of The Radiation Therapy Oncology Group (RTOG) 0225 or 0615. Radiotherapy-induced brain necrosis is one of the severe complications that can lead to cognitive dysfunction, seizure, headache, limb paralysis, and hematencephalon. Our study showed that besides lesion location, brainstem dose per unit volume, D0.1cc, and D1cc should also be considered. Moreover, for patients with microcirculation disturbance, such as diabetes, high blood pressure, and immune disorders, radiotherapy dose-volume parameters should be strictly limited (6, 7).

Dysphagia was identified as a major concern in NPC patients after a long-term follow-up. The incidence of dysphagia after radiotherapy for NPC has been reported to be 54%–95%, and dysphagia will continue to worsen with time (8, 9). Aspiration pneumonia due to dysphagia has become one of the leading causes of death after radiotherapy in patients with NPC (10, 11). The main factor of affecting swallowing function after radiotherapy was impaired swallowing structures (12), including the pharyngeal constrictors, vocal cords, upper larynx of the glottis, and upper esophagus (13). It has been demonstrated that radiotherapy can lead to anatomical changes and dysfunction of swallowing structures (14). In recent years, many studies have been conducted to explore treatment strategies to reduce swallowing dysfunction (15, 16). Researchers advocated reducing the target area V (50) of swallowing structures, but the maximal dose remained high due to surrounding dose coverage (17).

Factors influencing damage to swallowing structures include primary gross tumor and the lateral and medial group retropharyngeal lymph nodes. To date, the understanding of the primary lesions and the lateral group RLNs are relatively mature. The medial group RLNs are the blind spot due to its low incidence and the limitation of imaging technique. The medial group RLNs are close to the center line, located between the pharyngeal constrictor and vertebral front fascia (18). Sun Ying et al. proposed VIIc as the medial group RLNs (19). Since the medial group RLNs are currently recognized as high-risk areas for metastasis, researchers pointed out that no matter how big they were, they should be considered as malignant lesions (20). Because of its low incidence and limited imaging features, clinical characteristics of medial group RLNs are rarely reported.

So far, RTOG 0225/0615 protocols recommend the CTV60 delineation of VIIc, which cover the skull base to the superior border of hyoid bone. Nevertheless, there are controversies regarding the CTV60 boundaries of VIIc in clinical practices. Several cancer institutes have developed feasible guidelines to optimize the target volume of VIIc in China. For example, Sun Ying et al. pointed out that VIIc boundaries extended from skull base to the caudal edge of C2 (19). To date, no consensus guidelines or relevant clinical studies provide strong evidence for the delineation of VIIc boundaries in CTV60. VIIc has the pharyngeal constrictor muscle anteriorly and the long cephalic muscle posteriorly, with the medial border of the VIIa layer laterally and the midline medially (21). The main anatomical structures of VIIc are closely related to swallowing function. We conducted this pilot study to explore the clinical features of medial group RLNs and examine the feasibility of optimizing CTV60 of medial group RLNs from the skull base to the upper margin of C2 to reduce radiotherapy dose of swallowing structures.

## Materials and methods

### Study subjects

Patients received IMRT from June 2011 to February 2018 in Jiangsu Cancer Hospital. A total of 549 patients were included, and inclusion criteria were as follows: (1) with histologically proven NPC, pathologically diagnosed with non-keratinized undifferentiated carcinoma; (2) patients had not received antitumor treatment before biopsy sampling; (3) clinical stage I-IVa; (4) aged between 18 and 70 years; (5) with complete medical records and regular follow-up, without a history of cancer, and complete treatment. Exclusion criteria were as follows: (1) patients died of other diseases; (2) Other pathological types, such as adenocarcinoma and lymphoepithelial carcinoma; and (3) patients with second primary cancer. They underwent a comprehensive pretreatment evaluation. ^[18F]^Positron emission tomography and computed tomography (^[18F]^PET/CT) was performed when necessary. All patients were restaged according to the 8th edition of the American Joint Committee on Cancer (AJCC) staging system based on imaging materials and medical records. The institutional review board approved this study. The Clinical Research Ethics Committee of Jiangsu Cancer Hospital approved the protocol. This study was conducted in accordance with the Declaration of Helsinki. Informed consent was obtained from each participant.

### MRI examination

MRI examination was performed using Philips achieva1.5t superconducting MR scanner. Scanning sequences: (1) axial/coronal images: T1WI and STIR; (2) sagittal position: T1WI, T2WI. T1WI, and fat suppression were observed on axial, coronal, and sagittal T1WI after intravenous injection of Magnevist. DWI was performed before the injection of Magnevist, using single-excited spinal-plane echo and STIR sequences. Scanning parameters: The diffusion-sensitive factor b value was 1000s/mm^2^, and sensitive gradient pulses were applied to *x*, *y*, and *z* axes to obtain DWI in the 3D workstation. The apparent diffusion coefficient (ADC) images were generated using the software.

### Enhanced localization CT and frequent CT examination

A Philips Mx8000 multi-slice spiral CT was used. After intravenous injection of iohexol, patients underwent direct enhanced CT scanning. Frequent CT was defined as enhanced CT scans performed in fractions of 0, 5, 10, 15, and 25 during IMRT.

### Image analysis and diagnostic criteria

The images were analyzed independently by two head and neck image diagnostic specialists. The first step was to determine the presence of the medial group RLNs by two physicians on conventional T2 STIR scanning, DWI, and enhanced CT, respectively. The second step was to analyze the sensitivity among T2 STIR, DWI, and enhanced CT.

### Treatment

Prior to treatment, patients were immobilized with thermoplastic head and shoulder masks and underwent CT simulation according to standard procedures. MRI and fusion with simulation CT images were performed to assist target delineation. IMRT with 7–9 field fixed angle was adopted. Gross tumor volumes were defined based on MRI, CT, and PET/CT imaging before induction chemotherapy. The specific prescription doses were as follows: planned target area (PTVnx) of the primary tumor (GTVnx), 66–75 Gy for 32–34 times. PTVnd of metastatic cervical lymph node (GTVnd), 66–70 Gy, 32–34 times. PTV1 of CTV1 (high-risk area) and CTV2 (low-risk area) were 60.0 and 50.4 Gy, respectively. Dose limitations for organs at risk were described in detail previously (22). Institutional guidelines recommended only IMRT for stage I NPC and IMRT combined with concurrent chemoradiotherapy ± neoadjuvant/adjuvant chemotherapy for stages II–IVa NPC. Neoadjuvant/adjuvant chemotherapy regimens included TP (docetaxel 80 mg/m^2^ and cisplatin 80 mg/m^2^, day 1) and TPF (docetaxel 60 mg/m^2^ and cisplatin 60 mg/m^2^, day 1; fluorouracil 600 mg/m^2^/d, days 1–5) every 3 weeks for two to four cycles. Concurrent chemotherapy was weekly cisplatin (40 mg/m^2^) during IMRT. When possible, salvage treatments (surgery or chemotherapy) were provided for patients with documented relapse or persistent disease (23).

### Principles of optimizing the CTV60 of VIIc regional lymph nodes

The principle of optimizing the CTV60 delineation of the VIIc regional lymph nodes was from the base of the skull to the upper edge of C2 to protect the swallowing structures. The excluded cases were as follows: (1) When the nasopharyngeal lesion involved the oropharynx, the low-separation margin of CTV60 was set at 9–15 mm below the lesion; (2) when the VII regional lymph node was at or below the C1 level, the low separation margin of CTV60 was set at 3–6 mm below the lesion.

### ^[18F]^Fluorodeoxyglucose positron emission tomography/computed tomography

The 710 DiscoveryTM PET/CT scanner was obtained from GE Healthcare, and 18F-FDG reagent was provided by Nanjing JYAMS, Ltd. Before the examination, patients were asked to empty their stomachs for at least 6h prior to the blood sugar test. After the blood sugar level was confirmed, **^[18F]^** FDG was injected intravenously at the standard dose. Images were reconstructed after attenuation correction to obtain 3D CT, PET images, and PET/CT-blending images.

### Definition of positive medial RLNs

In the present study, we adopted the professor King AD’s definition for positive medial RLNs (20). The criteria for diagnosing positive medial group RLNs were the lymph nodes between the pharyngeal constrictor and the anterior vertebral fascia, regardless of their size.

### Dysphagia assessment and end points

This information was collected through telephone interviews. All patients were asked the following four questions about their swallowing disability. Do you have difficulties in (1) drinking; (2) eating; or (3) swallowing; (4) Do you cough when eating or drinking? The answers were recorded as “Yes” or “No”. Based on the answers to these questions, we constructed a study-specific categorical symptom score, DESdC (an acronym for difficulty drinking, eating, swallowing, and coughing while eating/drinking), to describe the presence of these symptoms. We also define five categories of DESdC scores ranging from 0 to 4. 0 = *no to all questions*; 1 = *yes to one question*; 2 = *yes to two questions*; 3 = *yes to any three questions*; and 4 = *yes to all four questions* (24).

### Follow-up visits

The follow-up period is from the first day of treatment to the last examination or the day of death. Patients were followed up every 3 months for the first 3 years after radiation therapy, every 6 months for the fourth to fifth years, and annually thereafter until death. Follow-up visits included physical examination, hematological and biochemical profiles, EBV-DNA, MRI, CT scan of the chest and abdomen, and whole-body bone scan. ^[18F]^PET/CT was performed when necessary. Local recurrence-free survival (LRFS) was defined as the time from the start of treatment to the first local failure. Progression-free survival (PFS) was defined as the time from initiation of treatment to failure or death from any cause, whichever occurred first. Distant metastasis-free survival (DMFS) was defined as the time from initiation of therapy to first distant failure. Overall survival (OS) was defined as the time from the initiation of therapy to death from any cause.

### Statistical methods

SPSS 24.0 software was used for statistical analyses and figures generation. Survival curves were depicted using the Kaplan–Meier method and compared by the log-rank test. The least absolute shrinkage and selection operator (Lasso) is a regression analysis method that performs both variable selection and regularization to improve predictive accuracy and statistical model interpretability. Lasso regression analysis was used to select highly correlated variables that were strongly associated with the occurrence of intermediate RLNs and survival indicators. Two-tailed *P* < 0.05 was considered statistically significant.

## Results

A total of 549 NPC patients with clinical stages I–IVa were included in the current study. Deaths caused by other diseases, such as secondary malignancies, cerebral hemorrhage, and liver injury after the use of herbal medicines, were excluded. Specific pathological patterns, such as sarcoma, myoepithelial carcinoma, adenocarcinoma, and mixed pathological patterns, were excluded.

**Figure 1:**
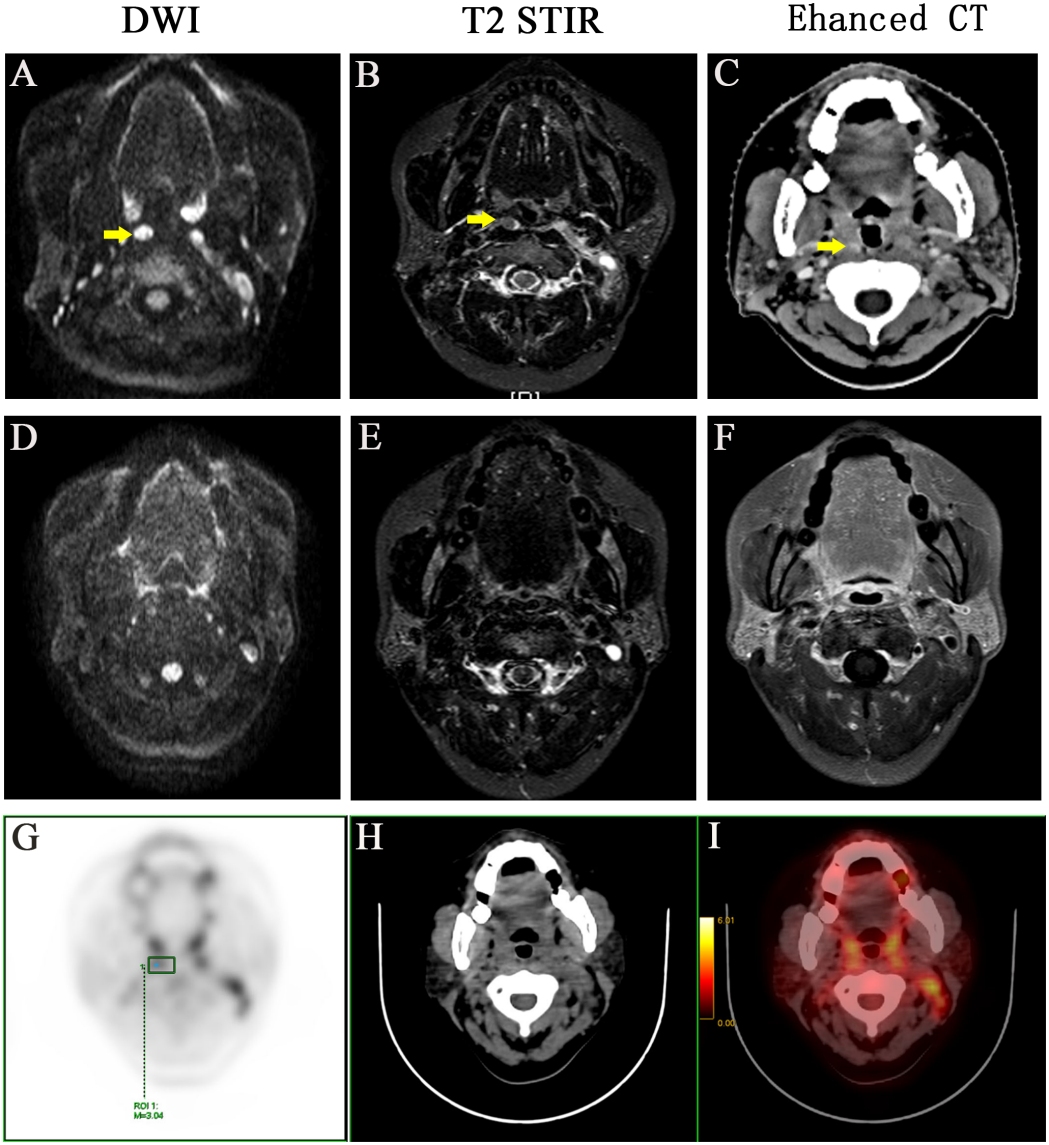
A representative case with positive medial group of retropharyngeal lymph node before and after treatment. Before treatment: **(A)** DWI imaging **(B)** T2 STIR **(C)** Enhanced CT. After treatment: **(D)** DWI imaging **(E)** T2 STIR **(F)** Enhanced CT. **(G-I)** PET-CT image before treatment, PET image, CT image, PET/CT blending image, respectively.

The medium follow-up months were 72 (5–129) months. Of the 549 patients, 14 (2.55%) had medial group RLNs with a transverse diameter of 2.0–19.0 mm. The percentages of those with transverse diameters of 2.0–5.0 mm and > 5 mm were 57.1% (8 of 14) and 42.9% (6 of 14), respectively. Eleven medial group RLNs were distributed between the upper edge of C1 and the upper 1 of 3 of C3. The sensitivities of DWI, T2 STIR and enhanced CT were 100%, 57.1%, and 21.4%, respectively (**Table 1**). DWI presented an absolute advantage in identifying small lymph nodes and the representative images are shown in **Figures 1A–C**.

**Table 1:** Comparison of DWI, T2 STIR and enhanced CT in the display of the medial group of retropharyngeal lymph nodes (n=14).

	Positive	Negative	Sensitivity (%)
DWI	14	0	100
T2 STIR	8	6	57.1
Enhanced CT	3	11	21.4

The incidence of medial group RLNs was not associated with age, gender, total clinical stage, T stage, anterior vertebral muscle invasion, or oropharyngeal invasion. The medial group RLNs were also unrelated to the lateral group RLN metastasis, lymph node necrosis, and extracapsular spread, local regional recurrence, or death in the lateral group (*P* > 0.05). However, their incidence were significantly associated with N stage (*P* = 0.021) and bilateral cervical lymph node metastasis (*P* = 0.015). In particular, the incidence of medial group RLNs was significantly higher when the transverse diameter of the patient’s cervical lymph nodes > 3 cm (*P* = 0.004), which deserved further clinical attention. In addition, the occurrence of medial group RLNs might also be significantly related to distant metastases (*P* = 0.003) (**Table 2**).

**Table 2:** The relationship between clinical characteristics and the medial group of retropharyngeal lymph node.

Clinical Characteristics	The medial group of retropharyngeal lymph nodes		P Value
	Negative,n=535 (%)	Positive, n=14 (%)	
**Age**			0.722
<50	280 (52.3)	8 (57.1)	
≥50	255 (47.7)	6 (42.9)	
**Gender**			0.751
Male	402 (75.1)	10 (71.4)	
Female	133 (24.9)	4 (28.6)	
**Clinical Stage**			0.538
I-II	155 (29.0)	3 (21.4)	
III- IV	380 (71.0)	11 (78.6)	
**T Stage**			0.837
T1- T2	177 (33.1)	5 (35.7)	
T3- T4	358 (66.9)	9 (64.3)	
**N Stage**			0.021*
N0- N1	317 (59.3)	4 (28.6)	
N2- N3	218 (40.7)	10 (71.4)	
**Anterior vertebral muscle invasion**			0.766
Yes	246 (46.0)	7 (50.0)	
No	289 (54.0)	7 (50.0)	
Oropharyngeal invasion			
Yes	30 (5.6)	1 (7.1)	
No	505 (94.4)	13 (92.9)	
**Bilateral retropharyngeal lymph nodes**			0.806
Yes	167 (31.2)	6 (42.9)	
No	368 (68.8)	8 (57.1)	
-Diameter>2cm			0.311
Yes	66 (12.3)	3 (21.4)	
No	469 (87.7)	11 (78.6)	
-Diameter>3cm			0.745
Yes	4 (0.7)	0 (0.0)	
No	531 (99.3)	14 (100)	
**Bilateral cervical lymph nodes**			0.015*
Yes	209 (39.1)	10 (71.4)	
No	326 (60.9)	4 (28.6)	
-Diameter>2cm			0.058
Yes	245 (45.8)	10 (71.4)	
No	290 (54.2)	4 (28.6)	
-Diameter>3cm			0.004*
Yes	100 (18.7)	7 (50.0)	
No	435 (81.3)	7 (50.0)	
**Lymph node necrosis**			0.239
Yes	152 (28.4)	6 (42.9)	
No	383 (71.6)	8 (57.1)	
**Lymph node fusion**			0.175
Yes	175 (32.7)	7 (50.0)	
No	360 (67.3)	7 (50.0)	
**Local regional recurrence**			0.725
Yes	27 (5.0)	1 (7.1)	
No	508 (95.0)	13 (92.9)	
**Distant metastasis**			0.003*
Yes	38 (7.1)	4 (28.6)	
No	497 (92.9)	10 (71.4)	
**Death**			0.717
Yes	54 (10.1)	1 (7.1)	
No	481 (89.9)	13 (92.9)	

*P-values were calculated using an unadjusted chi-square test.

Bold values means P<0.05.

**Figure 2:**
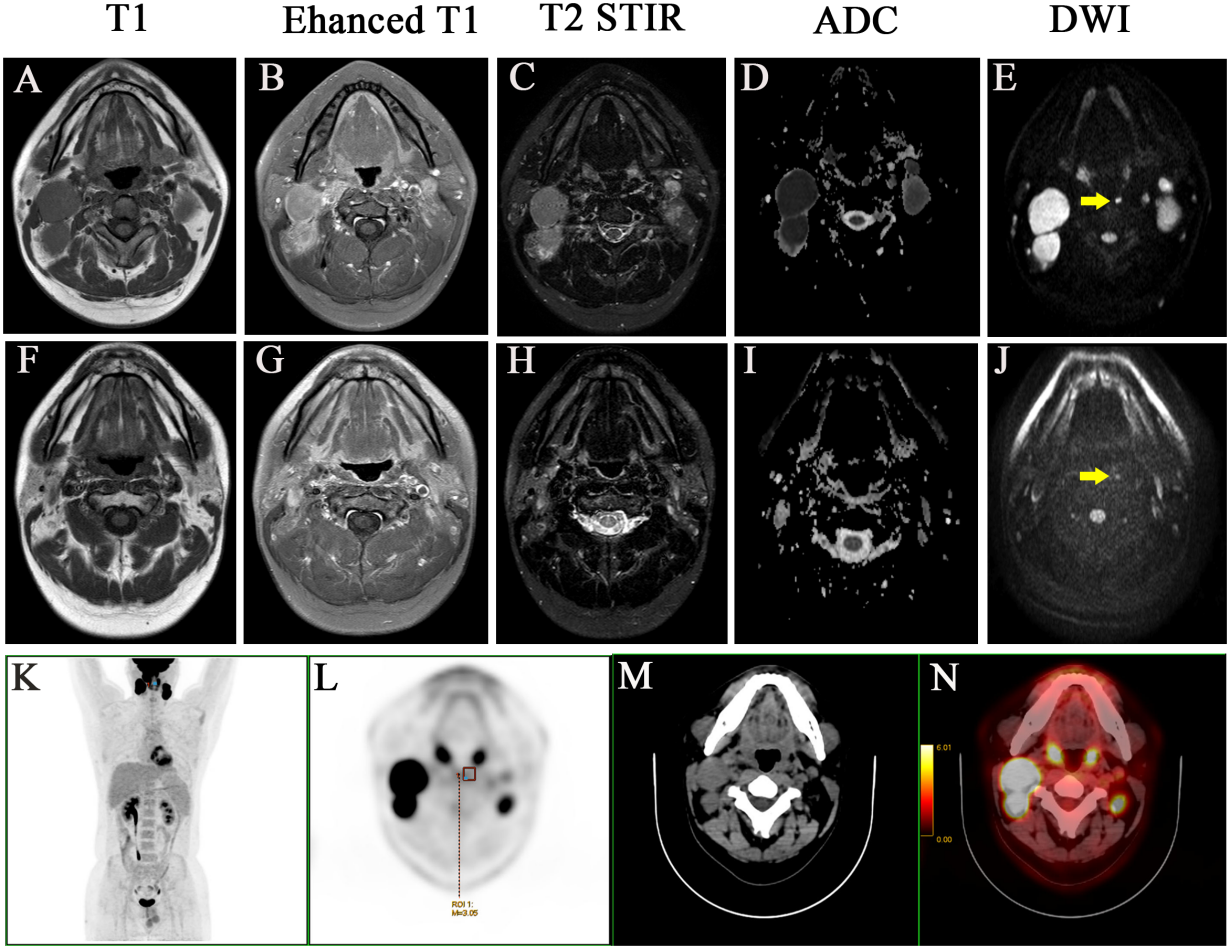
A case with the medial group of retropharyngeal lymph node did not retreat completely after treatment and two years follow-up. Before treatment: **(A)** T1 **(B)** Enhanced T1 **(C)** T2 STIR **(D)** ADC **(E)** DWI. After treatment: **(F)** T1 **(G)** Enhanced T1 **(H)** T2 STIR **(I)** ADC **(J)** DWI. **(K-N)** PET-CT image before treatment, MIP image, PET image, CT image, PET/CT blending image, respectively.

The lateral RLNs have been explored comprehensively and reached consensus guidelines. In this study, nine serial MRI scans were performed before and after radiotherapy to observe the medial group RLNs and evaluate the treatment responses of the medial RLNs. Thirteen of 14 (92.8%) cases showed completely regression after radiotherapy with or without concurrent chemotherapy; representative case was presented in **Figure 1** (before treatment: A–C; After treatment: D–F). Interestingly, one case slightly retreated after treatment, as shown in **Figure 2** (before treatment: A–E; after treatment: F–J). It was still identifiable, but the DWI signal was much weaker than before treatment (**Figures 2H, I**). ^[18F]^PET/CT was performed to detect FDG accumulation in tumor lesions. As shown in **Figures 1G–I**, lymph node with standardized uptake value (SUV) 3.04 was completely withdrawn, whereas the lymph node with SUV 3.05 was only reduced in size and DWI signal (**Figures 2K–N**).

**Figure 3:**
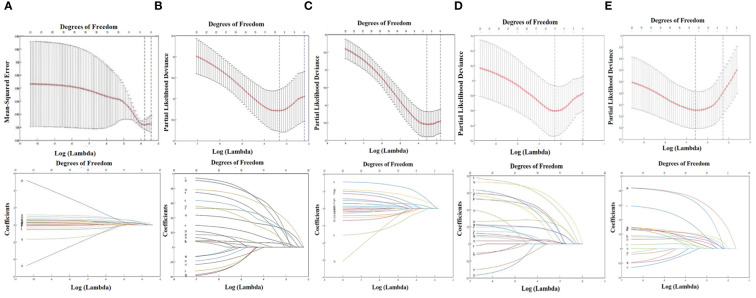
LASSO regression analysis about the medial group of retropharyngeal lymph node and survival indicators. **(A)** The relationship between the incidence of retropharyngeal lymph node and the clinical characteristics. **(B)** The relationship between OS and the clinical characteristics. **(C)** The relationship between LRFS and the clinical characteristics. **(D)** The relationship between DMFS and the clinical characteristics. **(E)** The relationship between PFS and the clinical characteristics.

Because of low incidence of the medial group RLNs, to avoid statistical discrepancy, we used Lasso regression analysis to select variables that related to the incidence of medial group RLNs. In addition to the clinical characteristics listed in **Table 2**, parotid lymph nodes and GTV dose were included in this analysis. The results showed that N stage, bilateral cervical lymph node metastasis, and the diameter of cervical lymph nodes > 3 cm were significantly associated with the incidence of medial group RLNs, which was consistent with the data from Pearson chi-square test (**Figure 3A** column). Meanwhile, we performed Lasso regression analysis to select factors for predicting prognosis. For OS, clinical stage, T stage, N stage, lymph node necrosis, and extracapsular spread, gender were crucial indicators (**Figure 3B** column). For LRFS, T stage, oropharyngeal invasion, the diameter of cervical lymph nodes > 2cm and lymph node necrosis were critical predicting markers (**Figure 3C** column). For DMFS, clinical stage, T stage, N stage, lymph node necrosis and extracapsular spread, oropharyngeal invasion, bilateral retropharyngeal lymph nodes, parotid lymph nodes, and radiation dose were relevant clinical features (**Figure 3D** column). For PFS, clinical stage, N stage, anterior vertebral muscle invasion, oropharyngeal invasion, lymph node necrosis and extracapsular spread, and parotid lymph nodes were identified as critical indicators (**Figure 3E** column).

**Figure 4:**
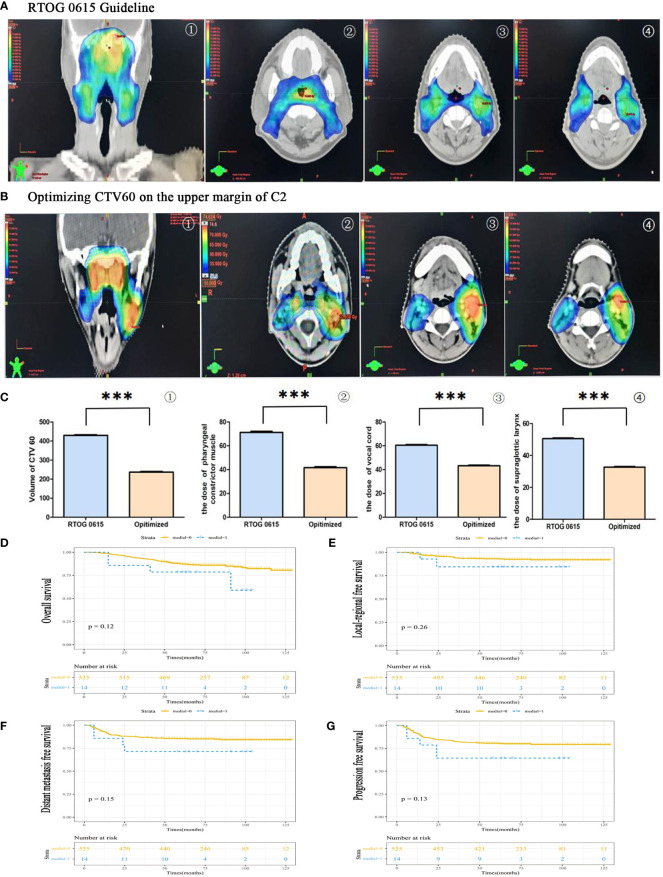
The feasibility of CTV60 optimization for VIIc regional lymph node from skull base to the upper margin of C2 to protect swallowing structures. **(A)** The group was delineated according to the protocol of RTOG 0615. The presented images were coronary side of the target volume ①, the layer of pharyngeal constrictor muscle ②, the layer of vocal cords ③ and the layer of supraglottic larynx ④, respectively. **(B)** The group was delineated according to our optimized strategy. The presented images were the coronary side of the target volume ①, the layer of pharyngeal constrictor muscle ②, the layer of vocal cords ③ and the layer of supraglottic larynx, respectively. **(C)** The statistical data of the volume of CTV60 ①, the dose of pharyngeal constrictor muscle ②, the dose of vocal cords ③ and the dose of supraglottic larynx ④. **(D)** Overall survival rate (OS). **(E)** Locoregional recurrence-free survival rate (LRFS). **(F)** Distant-metastasis-free survival rate (DMFS). **(G)** Progression-free survival rate (PFS). ***P<0.0005.

The clinical characteristics of medial group RLNs are crucial for CTV60 delineation. In this study, although a higher incidence of medial group RLNs (2.56%) was found than that in other studies, it was still rare, less than 5% in incidence. Our principles for optimizing CTV60 were discussed and got consistent agreements. The principle of CTV60 delineation for optimizing VIIc was from the skull base to the superior edge of C2 to protect swallowing structures. Our data show that the volume of high-dose radiotherapy (**Figure 4B①**), the dose and volume of the pharyngeal constrictor muscle (**Figure 4B②**), vocal cords (**Figure 4B③**), and supraglottic larynx (**Figure 4B④**) were significantly reduced compared with the protocol of RTOG 0615 (**Figures 4A①–④,C①–④**). We delineated one representative patient with RTOG 0615 and our optimized protocol (**Supplementary Figure S1**). The volume of CTV60 with optimized protocol was reduced significantly compared with RTOG0615 (230.2 cm^3^ vs 430.2 cm^3^) (**Supplementary Figure S1**). In addition, we measured the central dose at different typical transverse sections in terms of the upper margin of C2, epiglottis, superior, and inferior margin of hyoid in 50 NPC patients. The mean dose of the upper margin of C2, epiglottis, superior, and inferior margin of hyoid was 36.135, 30.881, 31.135, and 29.451 Gy, respectively (**Supplementary Figure S2**). For patients with oropharynx invasion and VII regional lymph nodes at or below the C1 level, we suggested a moderately extended boundary of CTV60 as follows. First, when the nasopharyngeal lesion involved oropharynx, the low separation margin of CTV60 was set at 9–15 mm below the lesion. Second, when the lymph nodes of VII region were at or below the C1 level, the low separation margin of CTV60 was set at 3–6 mm below the lesion.

For treatment-related toxicities, acute and late toxicities were assessed during treatment and long-term follow-up. Acute toxicities of radiotherapy and/or chemotherapy were well tolerated. Grades 1 and 2 acute mucositis, dermatitis, and xerostomia were most common. None of the patients discontinued the treatment course due to severe acute toxicity. During long-term follow-up, late toxicities were assessed by telephone interview. We asked patients if they had difficulties in drinking, eating, swallowing, or coughing while eating/drinking. We also scored the combined symptoms ranging from 0 to 4. Ninety-six of 549 patients had difficulties in drinking, eating, swallowing, or coughing when eating/drinking. Of the 96 patients, three patients had difficulty in drinking; 53 patients had difficulties when eating dry food, which can be released by drinking water; 10 patients had difficulties in swallowing; three patients had cough when eating or drinking; and three patients developed serious dysphagia because of its large tumor (**Table 3**). All of the 96 patients had difficulty in eating and 43 reported difficulty immediately after radiotherapy, which might be due to the damage of salivary glands. Eighty-three patients scored 1, seven patients scored 2, one patient scored 3, and three patients scored 4 (**Table 3**). The median time from onset of symptoms was 72 (4–114) months. Importantly, there was no regional recurrence in optimized area.

**Table 3:** Swallowing dysfunction evaluated with DESdC.

Category	Incidence (%)
DESdC symptoms	
Drinking Difficulties	3/549 (0.5)
Eating Difficulties	96 ^*^/549 (17.4)
Swallowing Difficulties	10/549 (1.8)
Coughing while Eating or Drinking	3/549 (0.5)
DESdC Score	
0	453/549 (82.5)
1	83/549 (15.1)
2	7/549 (1.3)
3	1/549 (0.2)
4	3/549 (0.5)

^*^ 43 of the 96 patients had difficulty immediately after radiotherapy, which might be due to the damage of salivary glands.

The 5-year OS rates in medial and non-medial groups were 78.6% and 87.3%, respectively (*P* = 0.12). (**Figure 4D**). The 5-year PFS rates in medial and non-medial groups were 64.3% and 80.5%, respectively (*P* = 0.26) (**Figure 4E**). The 5-year LRFS rates in medial and non-medial groups were 84.4% and 93.0%, respectively (*P* = 0.15) (**Figure 4F**). The 5-year DMFS rates in medial and non-medial groups were 71.4% and 85.3%, respectively (*P* = 0.13) (**Figure 4G**). The 5-year OS, PFS, LRFS, and DMFS for all enrolled patients were 87%, 80%, 93%, and 85%, respectively.

## Discussion

Commonly, nasopharyngeal cancer (NPC) is treated with high-dose radiotherapy in the clinic. Researchers have emerged to standardize tumor target delineation variations and guide dose prioritization for NPC radiotherapy. Special efforts are required in the proactive sparing of normal structures to minimize the incidence and severity of radiation-associated complications, many of which may pose lifelong detriments to the life quality in NPC patients. Severe swallowing structures dysfunction might lead to dysphagia (25). Previous study proposed that V60 greater than 12% of the inferior pharyngeal constrictor was significantly associated with increased rate of dysphagia which demanded enteral nutritional supporting treatment (26). Dysphagia after radiotherapy has been reported in NPC cohorts (27). Emerging studies showed that dysphagia had a significantly detrimental effect on health-related quality of life. Although the exact incidence of dysphagia is uncertain, some investigators have suggested that up to 50% of patients undergoing chemoradiotherapy for head and neck cancer may experience long-term dysphagia. It not only significantly diminished patients’ quality of life but it might also result in severe pulmonary complications, which might be a major cause of death (28, 29). In this study, we illustrated the clinical characteristics of medial group RLNs. Meanwhile, based on the primary tumor and VII regional lymph node, we proposed the optimization of CTV60 delineation to protect swallowing structures.

This study revealed that the medial group RLNs were generally small, and 57.1% of them were with a transverse diameter of < 5 mm, which distributed between the upper edge of C1 and upper third of C3. Some scholars suggested that the incidence of medial group RLNs was 0.3% (19), whereas others suggested that the incidence was 0.2% (30). The conclusions were all based on conventional MRI imaging. Of note, even conventional MRI imaging is capable of discriminating soft tissue, but detecting small lymph nodes remains a challenge (18). We made use of multi-modal imaging approaches including contrast-enhanced CT, MRI, and ^[18F]^PET-CT, which offered the most comprehensive anatomic depiction of tumor extent. These diagnostic images can be imported into radiation treatment planning systems and registered with simulation scans to facilitate contouring target volumes. DWI is a technology of MRI functional imaging systems. The basis of DWI is the diffusion motion of water molecules. At a certain value of b, the diffusion motion of water molecules is measured to predict changes of internal microstructure state. At a low b value, DWI is not sensitive enough to the diffusion of water molecules, and is easily affected by T2 penetration effect. With the increase of b value, the diffusion weight and contrast of DWI increase, and the sensitivity of small lymph nodes is improved. Based on the imaging of DWI, our results showed that the incidence of medial group RLNs was nearly 3%, which was much higher than previous studies (31). Among DWI, T2 STIR and enhanced CT, the sensitivity of DWI is least affected by the size of lymph nodes, rendering DWI an advantage in detecting small lymph nodes. We also performed ^[18F]^PET-CT to identify and ascertain the SUV of medial group RLNs. However, it did not show obvious advantage in presenting the small lymph nodes with diameter < 5 mm. PET-CT has its limitations, which can recognize lesions larger than 4 mm. When lesions are less than 4 mm, its diagnostic accuracy decrease obviously. In addition, the size limitation, researchers demonstrated that PET-CT imaging might also show false-positive lymph node due to concurrent infection (32). Based on the sensitivity and location of DWI, it showed high FDG uptake in medial group RLNs, which could help to distinguish the essence of the lesion.

According to the results of the Pearson chi-square test and Lasso regression analysis, the incidence of medial group RLNs was associated with N stage and bilateral cervical lymph node metastasis, especially when the transverse diameter of cervical lymph nodes was > 3 cm. We also performed Lasso regression analysis to select factors for predicting prognosis. For OS, clinical stage, T stage, N stage, lymph node necrosis, and extracapsular spread, gender were crucial indicators. For LRFS, T stage, oropharyngeal invasion, diameter of cervical lymph nodes > 2 cm and lymph node necrosis were important predicting markers. For DMFS, clinical stage, T stage, N stage, lymph node necrosis and extracapsular spread, oropharyngeal invasion, bilateral RLNs, parotid lymph nodes, and radiation dose were involved. For PFS, clinical stage, N stage, anterior vertebral muscle invasion, oropharyngeal invasion, lymph node necrosis, and extracapsular spread and parotid lymph nodes were critical indicators. Based on results from Lasso regression analysis, the medial group RLNs did not act as an independent prognostic indicator. All the above indicators may help us to construct predictive models that can be used to classify patients into different risk for individual treatment.

Lam and King suggested that the medial group RLNs should be considered as malignant lesions (18, 20). In this study, we found that 92.86% (13 of 14) of the cases showed complete regression following treatment while the remaining one only showed partial regression. The lymph node was present continuously during 3 years follow-up, which might suggest that the medial group RLNs might not be indicator of malignant lesions.

RTOG 0225/0615 are main guidelines for the delineation of NPC. Compared with RTOG 0225, RTOG 0615 mainly reduced the target volume of CTV anterior boundary and posterior boundary. Moreover, considering the low incidence of Ib region lymph nodes (about 3%), the revised guideline proposed that only positive lymph node should be irradiated (33). However, there were no changes about CTV60 of medial group RLNs, which was from the skull base to the superior margin of hyoid. Optimizing CTV60 of medial group RLNs in NPC patients are favorable in reducing swallowing dysfunction. In this study, we optimized CTV60 of medial group RLNs from skull base to the upper margin of C2 to protect swallowing structures, except two special lesions as described previously. Using this strategy, the dose and volume of pharyngeal constrictor muscle, vocal cords, and the glottis, especially the volume of high dose radiotherapy, were significantly reduced compared with the protocol of RTOG 0615. The 5-year OS, LRFS, DMFS, and PFS rates in medial group and no medial group showed no significant differences. The 5-year OS, LRFS, DMFS, and PFS survival probabilities of enrolled patients were 87%, 80%, 93%, and 85%, respectively, which was consistent with previous survival rates (1, 23, 34–36). None of regional recurrence occurred within optimized target region. Excellent outcomes supported the feasibility of individualized CTV60 delineation. Our result reached the consistent conclusion compared with a multicenter randomized phase 3 trial initiated by Jun Ma, in which all patients was 1:1 assigned to sparing group and standard group (37). Their conclusion included the following: (1) There were no significant difference in terms of 3-year OS, DMFS, and LRFS; (2) the acute and long-term side effect in sparing group were better than standard group, and patients could have a much better quality life. However, they did not clarify which patients could benefit from the sparing radiotherapy and which patients could undergo this kind of target optimization. Also, they randomised the patients 1:1 to two groups, which might ignore the individual clinical features as the NPC was of high heterogeneity. To some extent, our results provide supplementary clinical data to this clinical research. We not only clarified the characteristics of the medial group RLNs but also proposed individualized CTV60 delineation approach in different subtypes.

Compared with standardized treatments, all enrolled patients had a considerably improved quality of life both during and after therapy. Acute toxicities during radiotherapy were well tolerated. Ninety-six of 549 (17.5%) patients had symptoms in terms of difficulties in drinking, eating, swallowing, or coughing when eating/drinking. Eighty-three patients scored 1, seven patients scored 2, one patient scored 3, and three patients scored 4. Related symptoms occurred in 43 patients immediately after radiotherapy, which might due to the damage of salivary glands. The median interval from onset of symptoms in the rest 53 patients was 72 (4–114) months. To visualize dosimetric changes in target volumes and OARs, we performed CT scans on fractions of 0, 5, 15, and 25 throughout radiotherapy. We were able to not only replan the target volume according to the altered body contour and shifting tumor position but also monitor the optimization’s correctness and safety.

In summary, our result showed much higher incidence of medial group RLNs based on multi-model imaging. We should combine clinical features and multi-imaging records to comprehensively identify the essence of medial group RLNs. Considering the low incidence of medial group RLNs, optimizing CTV60 for VIIc from skull base to the upper edge of C2 is safe and feasible. This study had longer follow-up time and much more concrete optimized strategy of medial group RLNs, which might supplement the findings of the prospective clinical trial NCT03346109 (37). Moreover, this study clarified the advantage of DWI in presenting small lymph nodes. This study have several limitations, such as deficient number of cases, single-center retrospective study, and unvalidated questionnaires. As a single-center retrospective study, the results of this study need relevant prospective studies to verify. With increased awareness of long-term radiation complications and the advances in chemoradiotherapy, researchers will strive to reduce radiation dose and volume to improve the quality of life in NPC.

## Data availability statement

The raw data supporting the conclusions of this article will be made available by the authors, without undue reservation.

## Ethics statement

The studies involving humans were approved by the Clinical Research Ethics Committee of Jiangsu Cancer Hospital. The studies were conducted in accordance with the local legislation and institutional requirements. The participants provided their written informed consent to participate in this study.

## Author contributions

DZ and NJ collected the clinical data and wrote the manuscript. CK guided the statistical analysis. JW, L-JW, and Y-SG read images and delineated the CTV 60. L-FZ read images and helped finish data acquisition. XH read images and revised the manuscript. Z-ZC and S-FH designed and defined the intellectual content. All authors contributed to the article and approved the submitted version.
