# Supplementary figures and images for: Distribution pattern of medial group retropharyngeal lymph nodes and its implication in optimizing clinical target volume in nasopharyngeal carcinoma
Source: Front Oncol. 2023 Sep 5;13:1228994. doi: 10.3389/fonc.2023.1228994 (PMC10509553; doi:10.3389/fonc.2023.1228994)

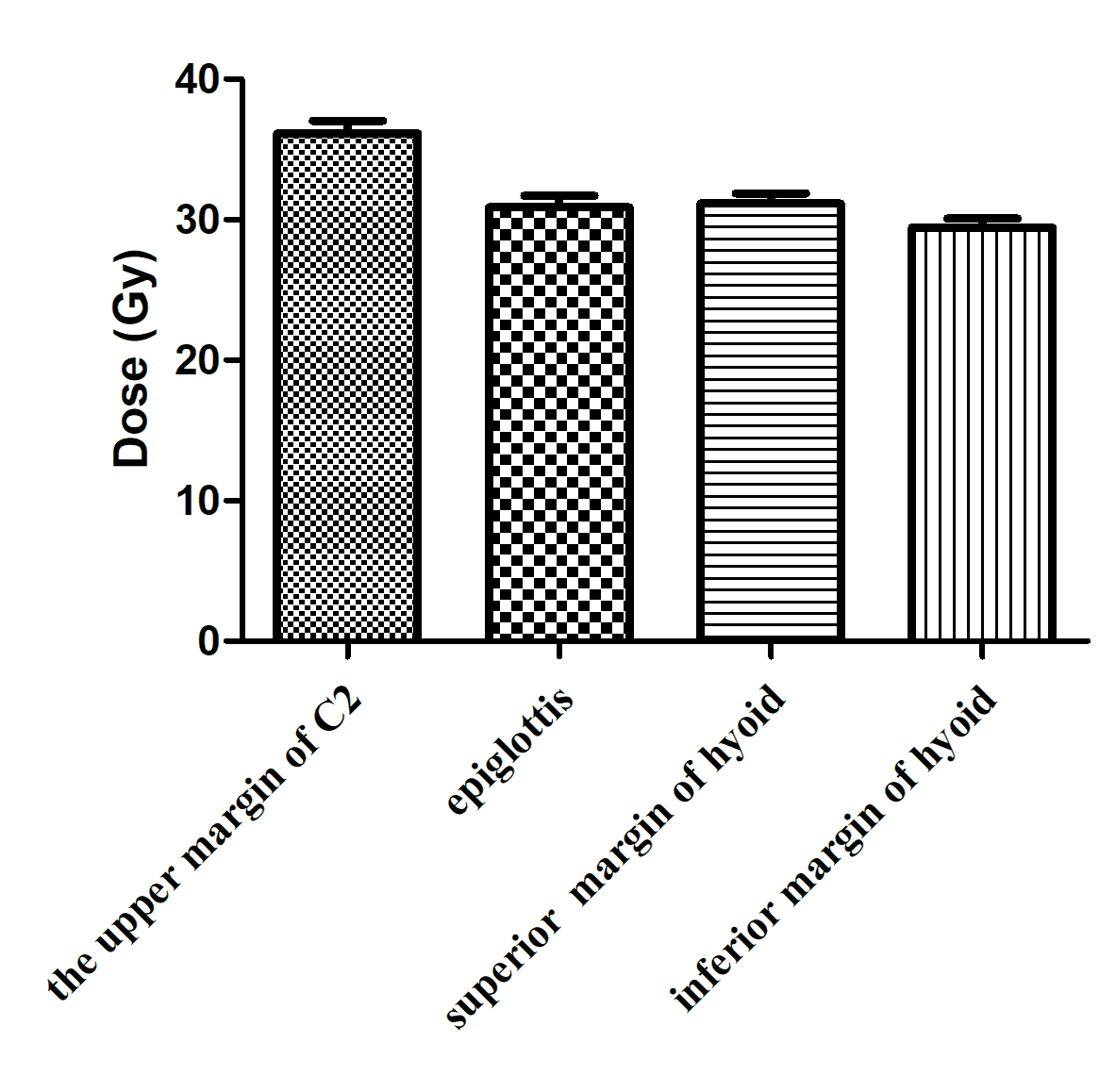

Supplement: Supplementary file 1 [file Image_1.tif]
