# Supplementary material for: Distribution pattern of medial group retropharyngeal lymph nodes and its implication in optimizing clinical target volume in nasopharyngeal carcinoma
Source: Front Oncol. 2023 Sep 5;13:1228994. doi: 10.3389/fonc.2023.1228994 (PMC10509553; doi:10.3389/fonc.2023.1228994)
Supplement: Supplementary file 2 [file Table_1.docx]

| **Clinical Characteristics Patients (N=549)%** |
| --- |
| **Gender**  Male 413（75.2）  Female 136（24.8）  **Age（year）**  <50 288（52.5）  ≥50 261（47.5）  **Follow (month)**  Average(range) 42.54 (6-94)  **Pathology type**  Non-keratinizing squamous carcinomas 549 （100.0）  **T stage**  T1-T2 180 (32.8)  T3-T4 369 (67.2)  **N stage**  N0-N1 320 （58.3)  N2-N3 229 (41.7)  **Clinical stage**  Ⅰ-Ⅱ 158 (28.8)  Ⅲ-Ⅳ 391 (71.2)  **Loco-regional recurrence**  Yes 32 (5.8)  No 517 (94.2)  **Distant metastasis**  Yes 72 (13.1)  No 477 (86.9)  **Death**  Yes 55 （10.0）  No 494 (90.0) |
