# Supplementary material for: Distribution pattern of medial group retropharyngeal lymph nodes and its implication in optimizing clinical target volume in nasopharyngeal carcinoma
Source: Front Oncol. 2023 Sep 5;13:1228994. doi: 10.3389/fonc.2023.1228994 (PMC10509553; doi:10.3389/fonc.2023.1228994)
Supplement: Supplementary file 3 [file DataSheet_1.pdf]

RTOG0615 Guideline

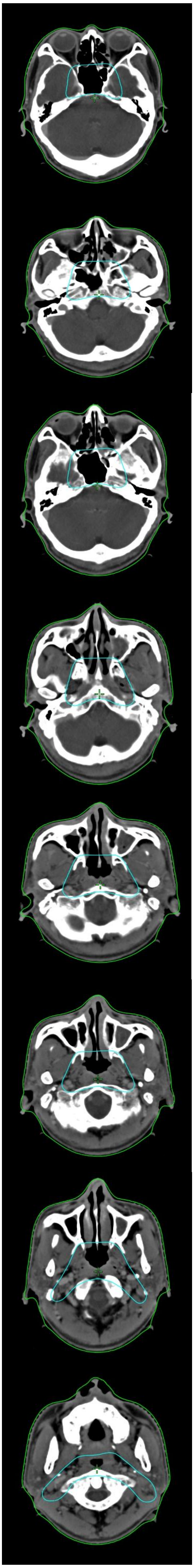

Optimizing CTV60 on the upper margin of C2

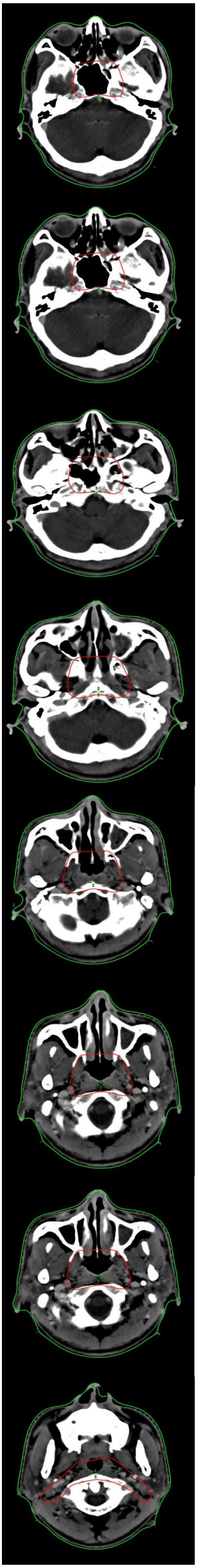

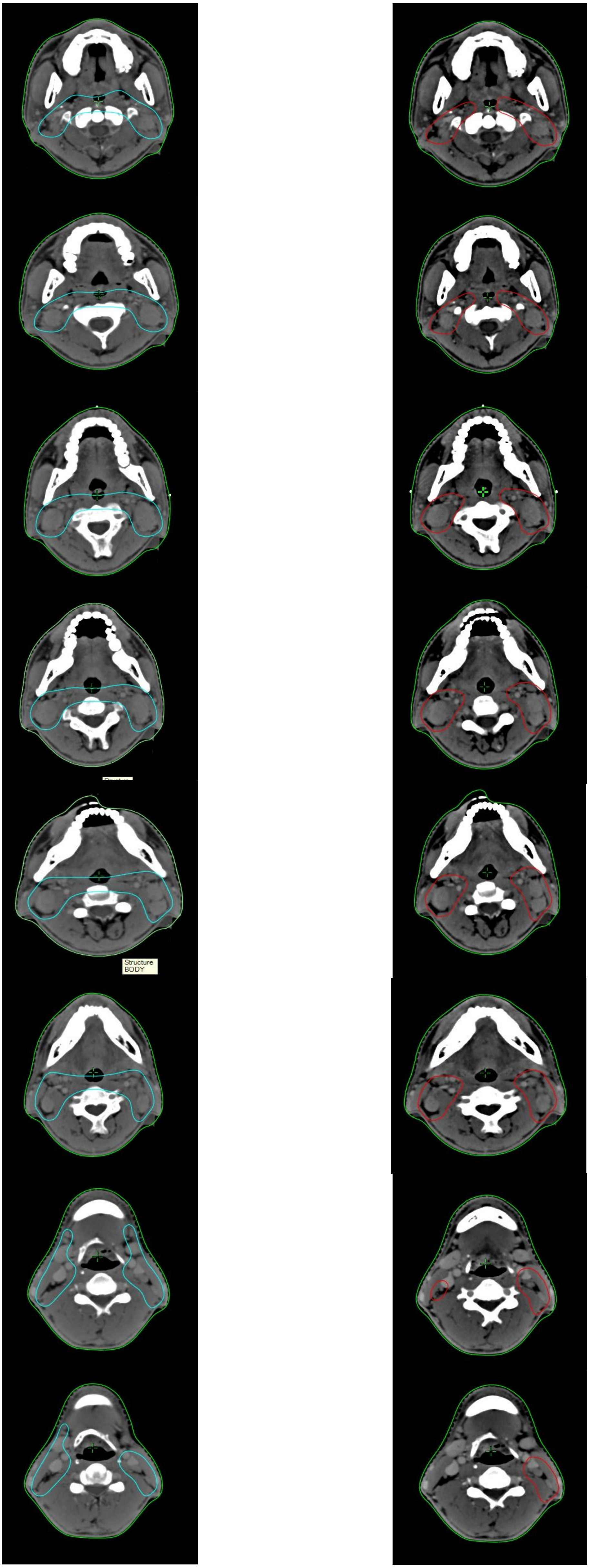
Structure
BODY

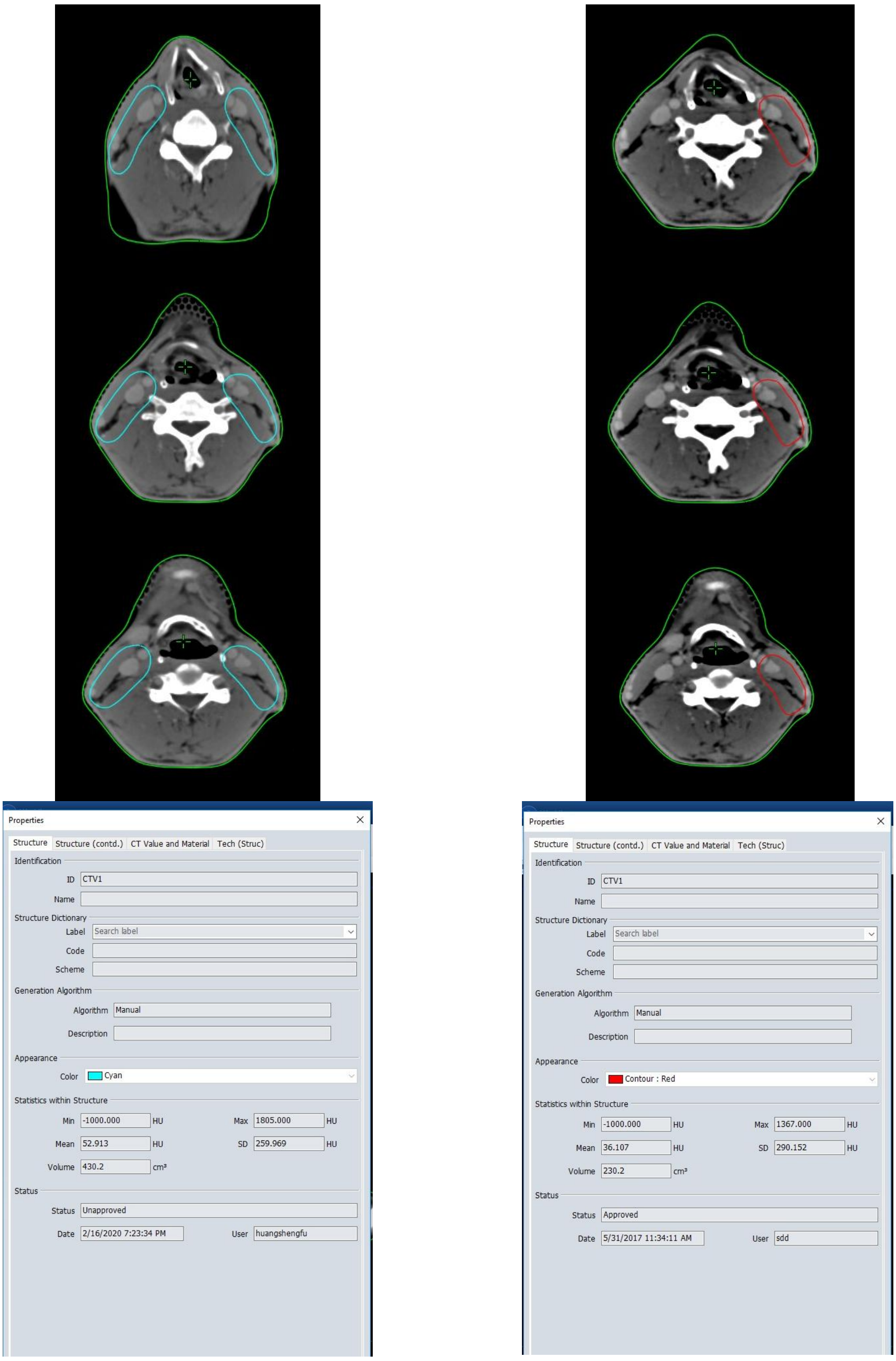
Properties
Structure
Structure (contd.)
CT Value and Material
Tech (Struc)
Identification
ID CTV1
Name
Structure Dictionary
Label Search label
Code
Scheme
Generation Algorithm
Algorithm Manual
Description
Appearance
Color Cyan
Statistics within Structure
Min -1000.000 HU
Max 1805.000 HU
Mean 52.913 HU
SD 259.969 HU
Volume 430.2 cm³
Status
Status Unapproved
Date 2/16/2020 7:23:34 PM
User huangshengfu
Properties
Structure
Structure (contd.)
CT Value and Material
Tech (Struc)
Identification
ID CTV1
Name
Structure Dictionary
Label Search label
Code
Scheme
Generation Algorithm
Algorithm Manual
Description
Appearance
Color Contour : Red
Statistics within Structure
Min -1000.000 HU
Max 1367.000 HU
Mean 36.107 HU
SD 290.152 HU
Volume 230.2 cm³
Status
Status Approved
Date 5/31/2017 11:34:11 AM
User sdd
